# Situs Inversus Totalis: Challenges and Anatomical Considerations in Endoscopic Retrograde Cholangiopancreatography

**DOI:** 10.7759/cureus.87427

**Published:** 2025-07-07

**Authors:** Usman I Aujla, Ahmad Karim Malik, Abdullah Saeed, Kashif Rafi, Imran Ali Syed

**Affiliations:** 1 Gastroenterology and Hepatology, Pakistan Kidney and Liver Institute and Research Center, Lahore, PAK; 2 Radiology, Pakistan Kidney and Liver Institute and Research Center, Lahore, PAK

**Keywords:** cholangiocarcinoma, common bile duct, endoscopic retrograde cholangiopancreatography, reversed anatomy, situs inversus totalis

## Abstract

Situs inversus totalis (SIT) is a rare condition characterised by the reversed positioning of abdominal and thoracic viscera. The anomaly poses a significant anatomical challenge during routine endoscopic procedures, including endoscopic retrograde cholangiopancreatography (ERCP). Here, we present the case of a 51-year-old patient with SIT and obstructive jaundice due to a periampullary mass. Initial ERCP attempts at an external facility for biliary decompression were unsuccessful, prompting referral to our center. Multidisciplinary consensus recommended preoperative ERCP followed by a Whipple’s procedure. ERCP was performed with positional adjustments (prone position) of the patient and significant scope manipulation (stepwise 360-degree anticlockwise rotation) to navigate the reversed anatomy. Cannulation was achieved, and a plastic biliary stent was placed, resulting in effective drainage. The patient demonstrated clinical improvement and was referred for surgical intervention. A comprehensive understanding of the reversed anatomy, along with the operator's skill and experience, is essential to address the challenges posed by this unique anatomical variation.

## Introduction

Situs inversus totalis (SIT) is a rare congenital anomaly in which the thoracic and abdominal viscera are positioned in a mirror-image pattern along the sagittal plane, opposite to their natural anatomical location [1]. The reported incidence of this rare entity is 1 in 8,000-25,000, with a higher frequency (3:2) among males [1,2]. The condition was first described in humans during the 17th century, when Fabricius reported the transposition of the liver and spleen [1]. In SIT, major organs such as the lungs, heart (presenting as dextrocardia), stomach, liver, and spleen are flipped to the opposite side of the body; however, they typically maintain normal organ function and vascular anatomy.

The embryological origin of SIT is linked to a defect in left-right axis patterning during early embryogenesis. This critical aspect of embryogenesis is orchestrated through a coordinated network of molecular signals involving nodal cilia. Genetic mutations (*DNAH5, DNAI1, *and* ZIC3*) have been linked to the development of SIT, particularly in association with primary ciliary dyskinesia (PCD). Approximately 50% of individuals with PCD exhibit SIT, reflecting a randomisation of laterality during development. Therefore, PCD has emerged as a key contributor to laterality disorders, given its strong association with defects in left-right axis determination during early embryogenesis [1,3].

While isolated SIT often remains asymptomatic and is diagnosed incidentally, its coexistence with PCD gives rise to Kartagener’s syndrome, a well-recognised triad marked by situs inversus, sinusitis, and chronic bronchiectasis. Individuals suffering from this syndrome present with recurrent ear and respiratory tract infections, male infertility, and an altered sense of smell. Other syndromes associated with abnormal positioning include asplenia, polysplenia, and Ivemark's syndrome [1,4]. The diagnosis of SIT can be established through detailed clinical assessment and radiological imaging. Initial radiologic assessment typically involves chest X-ray and abdominal ultrasound, whereas computed tomography (CT) and magnetic resonance imaging (MRI) offer detailed cross-sectional imaging for comprehensive anatomical evaluation [1].

Its diagnosis is critical in emergency conditions like polytrauma, acute cholecystitis, acute appendicitis, splenic injury, and acute myocardial infarction, as the abnormal sites of symptoms may lead to a delay in diagnosis and management [1,5-7]. Additionally, the condition poses a significant challenge for surgical and endoscopic interventions with standard indications. Particularly, the reversal of viscera makes procedures like endoscopic retrograde cholangiopancreatography (ERCP) and sphincterotomy technically difficult and complex compared to normal anatomical variants [8]. Here, we describe a case of a 51-year-old male with SIT who underwent ERCP for obstructive jaundice due to a periampullary mass.

## Case presentation

A 51-year-old male with a known history of ischemic heart disease and prior percutaneous coronary intervention presented with jaundice and pruritus lasting four weeks. Liver function tests demonstrated a cholestatic pattern, with a total bilirubin of 36 mg/dL, alkaline phosphatase of 496 U/L, and gamma-glutamyl transferase (GGT) level of 286 U/L. Initial imaging performed at an external facility included magnetic resonance cholangiopancreatography (MRCP), which revealed a soft tissue mass at the ampulla of Vater, resulting in proximal biliary dilatation. Endoscopic ultrasound (EUS) identified a 2.1 × 2.5 cm hypoechoic mass in the pancreatic head, causing retrograde biliary dilatation. A tissue biopsy confirmed a moderately differentiated adenocarcinoma of the pancreatic head. ERCP was attempted at the referring center for biliary decompression but was unsuccessful.

**Figure 1:**
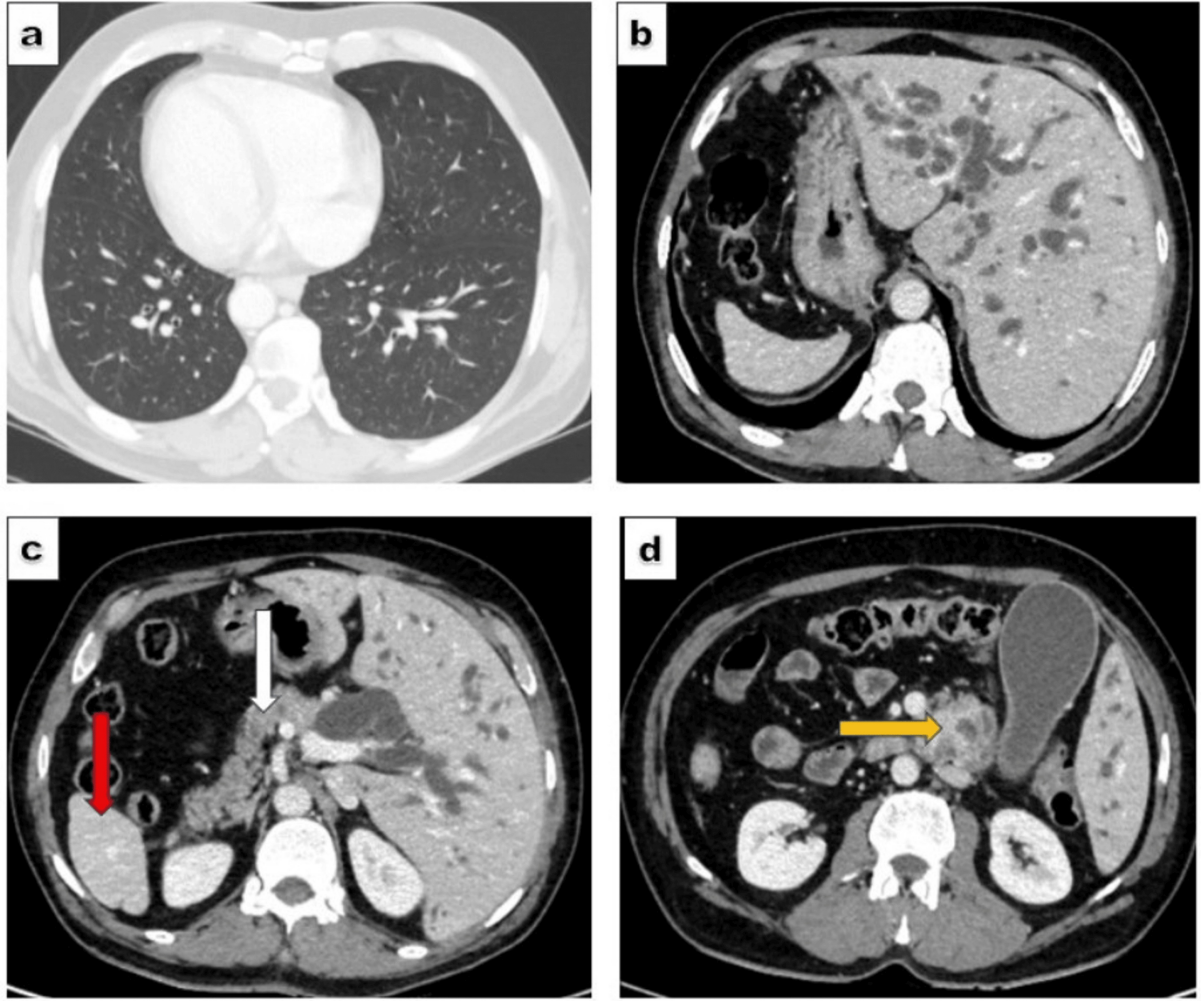
Computed tomography scan images of reversed anatomy. (a) Dextrocardia, (b) Liver on the left side, whereas the stomach lies on the right side, (c) Pancreas (white arrow) and spleen (red arrow) rotated on the right side, (d) Periampullary mass (yellow arrow).

The patient was subsequently referred to our institute for further management. A triphasic contrast-enhanced CT scan demonstrated complete situs inversus, characterised by reversal of the thoracic and abdominal viscera (Figure 1a-1c). A lobulated, heterogeneously enhancing mass measuring 4 × 3.6 cm was noted at the ampulla, resulting in moderate upstream biliary dilatation (Figure 1d). The regional mesenteric vessels were intact with preserved fat planes.

**Figure 2:**
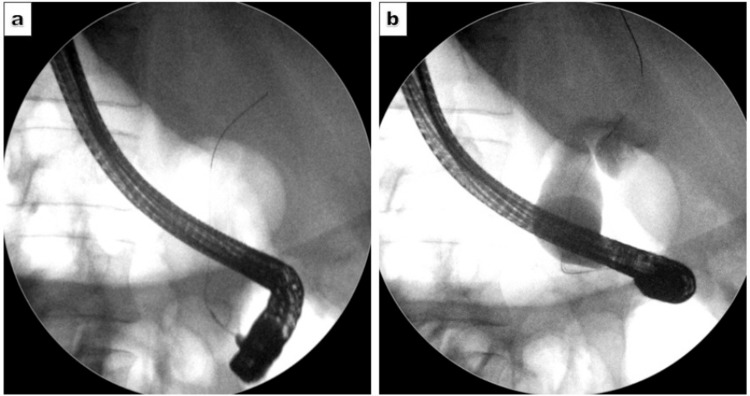
Cholangiogram. Cholangiogram showing (a) successful cannulation of the common bile duct and (b) contrast opacification reveals dilated common bile duct.

A multidisciplinary team recommended surgical resection via the Whipple procedure, with preoperative biliary drainage through ERCP. The ERCP was initiated with the patient in the left lateral decubitus position; however, due to difficulty manoeuvring the endoscope, the patient was repositioned to supine. Scope advancement required a 180-degree anticlockwise rotation to traverse the stomach and intubate the pylorus. Further visualisation of the ampulla necessitated an additional 180-degree anticlockwise rotation with sustained torque. The ampulla appeared bulky and infiltrated, consistent with malignancy. Selective cannulation of the common bile duct (CBD) was achieved successfully, followed by biliary sphincterotomy (Figure 2).

**Figure 3:**
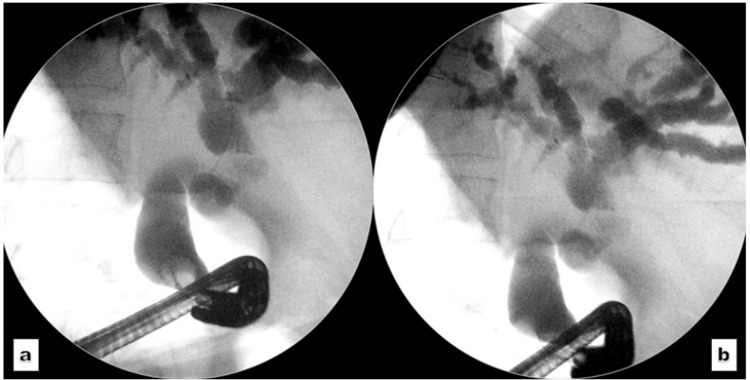
Occlusion cholangiogram. (a, b): Extreme angulation of the duodenoscope to obtain an occlusion cholangiogram, revealing a moderately dilated common bile duct and intrahepatic biliary channels in a liver with reversed anatomy.

A guidewire was advanced into the left hepatic duct. Occlusion cholangiography demonstrated rotated anatomy and a short distal CBD stricture, with marked dilatation of the CBD and intrahepatic ducts (Figure 3).

**Figure 4:**
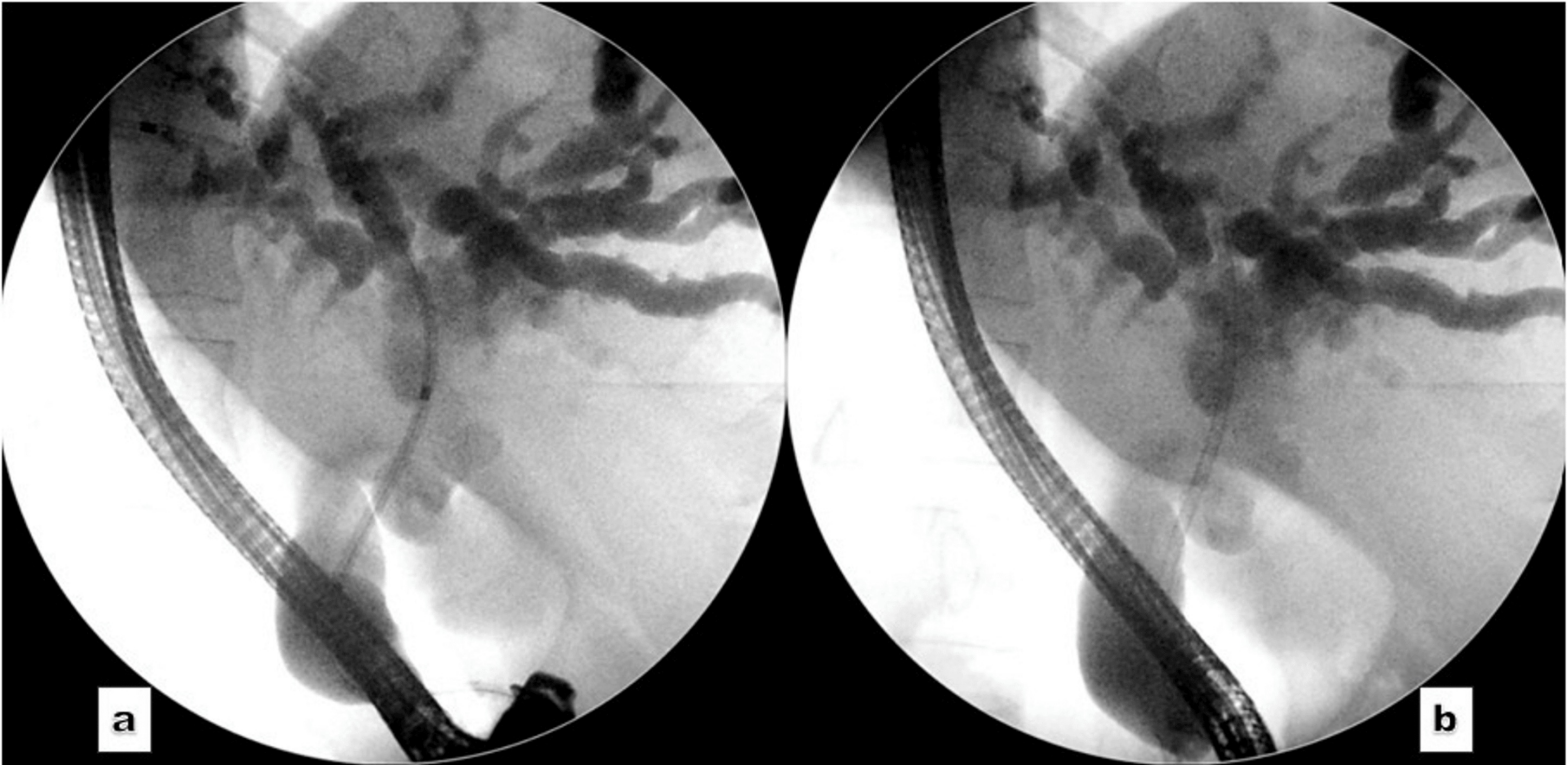
Cholangiogram. (a) The passage of the stent assembly and (b) the successful placement of a plastic biliary stent.

Biopsies and brushings were not repeated, as the diagnosis of adenocarcinoma had already been histologically confirmed. A 10 Fr × 12 cm plastic biliary stent was placed, achieving excellent biliary drainage (Figure 4).

Post-ERCP, there was a significant reduction in bilirubin levels, and the patient was referred to the surgical department for further management. Table 1 illustrates the pre and post-ERCP changes in liver function tests.

**Table 1:** Changes in liver function tests pre and post-ERCP. ERCP: endoscopic retrograde cholangiopancreatography.

Parameters	Unit	Pre-ERCP	Post-ERCP	Normal range
Total bilirubin	mg/dL	36.0	2.7	0.2-1.2
Alanine aminotransferase (ALT)	U/L	80	71	0-55
Aspartate aminotransferase (AST)	U/L	95	38	5-34
Alkaline phosphatase (ALP)	U/L	510	108	40-130
Gamma glutamyl transferase (GGT)	U/L	85	52	12-64

## Discussion

The evolution of ERCP has been a remarkable journey in the management of pancreaticobiliary disorders, transforming from a diagnostic entity into an effective therapeutic modality. The American Society for Gastrointestinal Endoscopy (ASGE) has stratified ERCP procedures into four complexity tiers, ranging from Level I (least complex) to Level IV (most complex). Performing a routine ERCP in surgically altered anatomy (e.g., Roux-en-Y or Billroth II anatomy) has been classified as a Level IV procedure by ASGE [9]. Similarly, the naturally altered anatomy encountered in SIT poses a significant challenge when performing ERCP for routine indications. The technical and clinical success of ERCP in these patients relies on a thorough understanding of the anatomical anomaly and the operator’s level of expertise. In this case, the patient had an unsuccessful ERCP attempt at an external facility and was referred to our specialised unit for the management of technically challenging and complex anatomy.

Among the major technical complexities, the reversed endoscopic view, altered fluoroscopic orientation, and direction of cannulation are the predominant limitations encountered during ERCP. To navigate these challenges, endoscopists must modify the conventional approach to gain access to the biliary and pancreatic ductal systems. Various techniques have been described in the literature to overcome these complexities. In the mirror image technique, the patient is placed in the right lateral decubitus position, while the endoscopist operates from the left side of the table, performing standard manoeuvres in reverse [10]. In the 180-degree clockwise rotation method, the patient is positioned in a left lateral or prone decubitus position, and the endoscopist stands on the right side of the table. The endoscope is rotated 180 degrees within the stomach or duodenum to reverse the usual direction of advancement and facilitate access to the ampulla and subsequent cannulation [8]. A 360-degree rotation technique has also been described in the literature, wherein an initial 180-degree rotation is performed in the stomach, followed by a second 180-degree rotation in the duodenum in the same direction. This approach helps overcome the challenge of reversed anatomy. However, the 360-degree turn technique presents difficulty in controlling the looped shaft and in cannulating the rightward-deviated ampulla [8].

Although no standardised protocol exists for ERCP in situs inversus totalis (SIT), expert consensus and case-based experience support a structured approach. Most procedures are performed in the prone position, with recognition that the ampulla lies on the mirror-image side. Successful cannulation often requires significant anticlockwise or clockwise rotation of the duodenoscope and precise scope torquing to align with the reversed anatomy. Standard cannulation tools are used, though rotatable sphincterotomes or guidewire-assisted techniques may aid in difficult cases. Operator familiarity with the altered spatial orientation and flexibility in technique is key to procedural success. In this case report, a stepwise anticlockwise 360-degree rotation (180 degrees in the stomach and another 180 degrees in the duodenum) of the endoscope, with the patient in a prone position, facilitated successful duodenoscope passage into the duodenum and allowed for effective biliary cannulation and stenting. 

Complications during ERCP in patients with situs inversus totalis (SIT), though uncommon, have been documented. One case series reported adverse events in 21.4% of patients (3 out of 14), including bleeding, pneumonia, and myocardial infarction. Another report described bleeding from portal biliopathy, successfully managed with stenting and balloon compression. Notably, patient positioning impacted outcomes-those in a modified position had higher cannulation success (90.9%) and fewer complications (18.2%) compared to those in the prone position (66.7% success, 33.3% complications), indicating a potentially increased risk with conventional positioning [11,12]. In the present case, ERCP was completed without any procedure-related complications, further underscoring the feasibility and safety of the intervention when performed with meticulous planning and expert technique.

## Conclusions

SIT presents a significant anatomical challenge during both diagnostic and therapeutic endoscopic procedures, such as ERCP and EUS. A comprehensive understanding of the reversed anatomy, coupled with the operator’s experience, is essential for effectively managing the procedural difficulties posed by this altered anatomical configuration. Formal grading of such complexity by international endoscopy societies is warranted to facilitate appropriate referrals and ensure optimal procedural planning and outcomes.
